# Biological Activity of Essential Oils of Four Juniper Species and Their Potential as Biopesticides

**DOI:** 10.3390/molecules26216358

**Published:** 2021-10-21

**Authors:** Ivanka Semerdjieva, Valtcho D. Zheljazkov, Tzenka Radoukova, Ivayla Dincheva, Neshka Piperkova, Vasilina Maneva, Tess Astatkie, Miroslava Kačániová

**Affiliations:** 1 Department of Botany and Agrometeorology, Agricultural University, 4000 Plovdiv, Bulgaria; v_semerdjieva@abv.bg; 2 Crop and Soil Science Department, Oregon State University, 3050 SW Campus Way, 109 Crop Science Building, Corvallis, OR 97331, USA; 3 Department of Botany and Methods of Biology Teaching, Faculty of Biology, University of Plovdiv Paisii Hilendarski, 4000 Plovdiv, Bulgaria; kiprei@abv.bg; 4 Plant Genetic Research Group, AgroBioInstitute, Agricultural Academy, 1164 Sofia, Bulgaria; ivadincheva@yahoo.com; 5 Department of Phytopathology, Agricultural University, Mendeleev 12, 4000 Plovdiv, Bulgaria; npiperkova@abv.bg; 6 Plant Protection and Technology Department, Institute of Agriculture, Karnobat, Agricultural Academy, 8400 Karnobat, Bulgaria; maneva_ento@abv.bg; 7 Faculty of Agriculture, Dalhousie University, Truro, NS B2N 5E3, Canada; astatkie@dal.ca; 8 Institute of Horticulture, Faculty of Horticulture and Landscape Engineering, Slovak University of Agriculture, 94976 Nitra, Slovakia; kacaniova.miroslava@gmail.com; 9 Department of Bioenergetics and Food Analysis, Institute of Food Technology and Nutrition, University of Rzeszow, 35-601 Rzeszow, Poland

**Keywords:** *Juniperus*, repellent activity, insecticidal activity, antifungal activity, antioxidant, antimicrobial activity, biopesticides

## Abstract

The objective of this study was to assess the biological activity of essential oils (EOs) of four *Juniperus* species obtained via two different distillation methods and their potential as biopesticides. The studied factors were juniper species (*Juniperus communis* L., *J. oxycedrus* L., *J. pygmaea* C. Koch., and *J. sibirica* Burgsd), plant sex (male (M) and female (F)), and distillation method (hydrodistillation via a standard Clevenger apparatus (ClevA) and semi-commercial (SCom) steam distillation). The hypothesis was that the EO will have differential antioxidant, antimicrobial, and insecticidal activities as a function of plant species, plant sex, and distillation method. The two distillation methods resulted in similar EO composition within a given species. However, there were differences in the EO content (yield) due to the sex of the plant, and also differences in the proportions of some EO components. The concentration of α-pinene, β-caryophyllene, δ-cadinene and δ-cadinol was dissimilar between the EO of M and F plants within all four species. Additionally, M and F plants of *J. pygmaea*, and *J. sibirica* had significantly different concentrations of sabinene within the respective species. The EOs obtained via ClevA extraction showed higher antioxidant capacity within a species compared with those from SCom extraction. All of the tested EOs had significant repellent and insecticidal activity against the two aphid species *Rhopalosiphum padi* (bird cherry-oat aphid) and *Sitobion avenae* (English grain aphid) at concentrations of the EO in the solution of 1%, 2.5%, and 5%. The tested EOs demonstrated moderate activity against selected pathogens *Fusarium* spp., *Botrytis cinerea*, *Colletotrichum* spp., *Rhizoctonia solani* and *Cylindrocarpon pauciseptatum*. The results demonstrate that the standard ClevA would provide comparable EO content and composition in comparison with SCom steam distillation; however, even slight differences in the EO composition may translate into differential bioactivity.

## 1. Introduction

Essential oils (EOs) are important natural products (NP) utilized in the development of new products, including environmentally safe pesticides [1]. They have shown significant biological activities such as antibacterial, antimicrobial, antifungal, antiviral and insecticidal, and play a notable role in allelopathic communication between plants [1,2,3,4,5,6]. Juniper species are important as a source of complex mixtures of secondary metabolites, especially EO. The main components of junipers oils are monoterpene hydrocarbons (α-pinene, β-pinene, *δ*-3-carene, and limonene) [7,8,9,10]. According to *Flora of Bulgaria* [11], in the Bulgarian flora, there are six species of genus *Jiniperus* (*J. excelsa*; *J. communis*; *J. oxycedrus*; *J. sibirica*; *J. sabina*; *J. pygmaea*). Two of them are of limited distribution (*J. excelsa; J. sabina),* but the others (*J. communis*; *J. oxycedrus*; *J. sibirica*; *J. pygmaea)* are widely distributed, and they have been known to encroach on pasture lands, especially in the mountains. For example, *J. communis* and *J. sibirica* have a dominant distribution, spreading intensively (and invasively) in most of the mountains of Bulgaria (Stara Planina, Rila, Pirin, Slavyanka) [12]. This intensive distribution of juniper species adversely affects the ecological balance of the ecosystems because: (1) the distribution of other plant species is restricted, and (2) the spread of junipers reduces the usable pastures. In some countries and states, they were declared unwanted species in such habitats. Many farmers are trying to limit the spread of *J. communis* and *J. sibirica* by means of various chemical and physical methods such as cutting or burning juniper bushes. On the other hand, junipers are potential resources for high-value EO. Currently, juniper EO has an expanding market; it is highly valued as an aroma agent and there is a significant production of juniper EO in Europe, North America, and Asia. Therefore, juniper removal can be coupled with juniper EO production. A literature review showed several biological activities of juniper EO, such as antibacterial, antifungal, antiviral, and antioxidant activities [7,8,9,10,13,14,15]. The biological activity of EOs is influenced by a number of factors, such as the composition of the EO, the distillation timeframe, the extraction method, and plant species [8,14,16,17,18,19]. *Juniperus communis* is one of the most widely distributed species on the planet [20], and therefore, among all juniper species, its EO composition has been reported most often. The antimicrobial activity of *J. communis* galbuli EO obtained with different distillation timeframes was demonstrated against *S. aureus* subsp. *aureus*, *C. glabrata* and *K. pneumonia* [14]. The antimicrobial activity of the EOs of fruits (galbuli/berries) and leaves (needles) of *J. communis* subsp. *hemisphaerica* and *J. oblonga* were investigated against *B. subtilis, S. aureus, E. coli, P. aeroginosa* and *C. albicans* [21]. Angioni et al. [13] tested the EOs and their major compounds of ripe and unripe berries and leaves of *J. oxycedrus* L. ssp. *oxycedrus*, *J. phoenicea* ssp. *turbinata* and *J. communis* ssp. *communis* against *C. albicans, S. aureus, E. coli,* and *P. aeruginosa* in Italy, and concluded that the EOs of *J. phoenicea* ssp. *turbinata* and the EO from leaves of *J. oxycedrus* ssp. *oxycedrus* exhibit either good or weak activity against *C. albicans* and *S. aureus*. The leaf EOs of M *J. communis, J. sibirica,* and *J. pygmaea* showed higher antimicrobial activity against *E. coli, H. influenzae, S. sonei, Y. enterocolitica, S. aureus* subs. *aureus, S. pneumonia*, compared to the EOs of female junipers [7].

Some EOs have the potential to scavenge free radicals of cells and may play an important role in the prevention of some diseases [22]. The antioxidant activity of the EOs from the juniper leaves and galbuli have been previously reported [14,23,24]. There are some previous reports on the composition of the EO from the berries and leaves of the *Juniperus* species; however, very few studies reported the antifungal and insecticidal activity of juniper EOs. The terpenoid compounds of EO of *J. saltuaria*, *J. squamata* var. *fargesii* and *J. squamata* var*. morrisonicola,* and *J. communis* were found to be highly toxic to insects [15,25,26]. Juniper EOs have the potential to be used as an ingredient in the development of new environmentally friendly insecticides, and therefore reduce the use of synthetic pesticides [1,10,15].

The objective of this study was to assess the antioxidant, antimicrobial, and insecticidal activities of M and F *J. communis*, *J. oxycedrus, J. pygmaea,* and *J. sibirica* EOs obtained by means of two distillation methods (ClevA and SCom). The hypothesis was that EOs of these four juniper species will have different antioxidant, antimicrobial, insecticidal and antifungal activities, and the standard method of ClevA hydrodistillation would provide dissimilar EO profiles when compared to a commercial steam distillation unit.

## 2. Results and Discussion

### 2.1. Total Essential Oil (EO) Content (Yield)

Overall, there was a substantial variation in the EO yield between M and F plants, plant species, and extraction methods (ClevA and SCom) (Table 1). The EO yield varied from 0.05% in *J. oxycedrus* (F) to 1.63% in *J. communis* (F). The highest yield of EO was obtained from *J. communis* (F) and *J. pygmaea* (F) with ClevA and SCom extraction (Table 1). Previous reports [27,28] have shown that the EO yield of *Juniperus* may depend on the gender of the plants, genetic characteristics, soil, and climatic factors. In this study, higher EO yields were obtained using ClevA in comparison with SCom distillation.

### 2.2. Composition of the Juniper Essential Oil (EO)

Gas chromatography (GC) and mass spectroscopy (MS) analyses of each of the four junipers EOs identified at least 35 constituents (Supplemental Tables S1–S4). Among the identified classes, the main ones were monoterpenes hydrocarbons (MH), oxygenated monoterpenes (OM), phenolic monoterpenes (PhM), bicyclic oxygenated monoterpenes (BOM), sesquiterpenes hydrocarbons (SH), oxygenated sesquiterpenes (OS), tricyclic oxygenated sesquiterpenes (TOS), bicyclic sesquiterpene hydrocarbons (BSH), oxygenated bicyclic sesquiterpenes (OBS) and diterpenes (D) (Table S5). The monoterpenes were the main class of EOs in all four junipers (Table S5).

As shown in Table 2, α-pinene was the predominant constituent of the monoterpenes in all four species (*J. communis*, *J. pygmaea, J. sibirica, J. oxycedrus*), with the highest concentration being in *J. communis* (F) EO. α-pinene varied from 14.9% (in *J. sibirica* F) to 34.9% (in *J. communis* F). The concentration of sabinene was generally above 9% of the EO, except in *J. oxycedrus* (M, F) (Table 2). Overall, the concentration of limonene was higher in *J. communis* (M) and *J. oxycedrus* (M) and lower in *J. sibirica* (M, F) (Table 3). α-terpinene, p-cymene, bornyl acetate, β-elemene were minor (below 2% of the EO) constituents in the EOs of all four species (Table 2 and Table 4). β-myrcene and terpinen-4-ol were found at higher concentrations in the EO of *J. communis* (Table 2 and Table 3). Overall, the monoterpenes in the EOs obtained via ClevA were slightly higher compared to those in the EO obtained through SCom. One sample of *J. communis*-M obtained through ClevA was an exception (Table S5).

In general, the values of sesquiterpenes in the four juniper species were higher in the EO obtained via SCom (Table S5). The monocyclic sesquiterpene hydrocarbons (MSH) and bicyclic sesquiterpene hydrocarbons (BSH) were the most represented of this class (Table S5). Germacrene D and β-elemene were the predominant compounds of the monocyclic sesquiterpene hydrocarbons (MSH). The highest amount of germacrene D was found in *J. oxycedrus* (17.8% F), followed by *J. sibirica* (13.1% M), *J. pygmaea* (11.4% F), and *J. communis* (6.9% M) (Table 4). β-elemene was higher in *J. sibirica* (F) EO (6.8%), followed by *J. pygmaea* (F) (4.8%) (Table 3). β-caryophyllene, γ-cadinene, δ-cadinene, and α-humulene were predominant among the bicyclic sesquiterpene hydrocarbons (BSH) (Table 3 and Table 4). The highest γ-cadinene values were obtained from *J. oxycedrus* (M, F), followed by *J. pygmaea* F (3.2%) (Table 4), while α-humulene was highest (2.98%) in *J. pygmaea* (F) EO. The compositions of *J. communis* EO extracted by SCom steam distillation were specific and contained δ-cadinol, tau-cadinol, tau-muurolol, α-cadinol. Acyclic sesquiterpene hydrocarbons were found only in *J. sibirica* (farnesol and farnesal) (Table S5).

The diterpenes class was the highest in *J. oxycedrus* (M) EO (~20%) (Table S5). Oxygenated diterpenes were found only in *J. oxycedrus* and *J. communis* EO, while monocyclic diterpenes were found in *J. sibirica* EO.

Generally, the EO composition of the respective samples obtained by the two extraction methods (ClevA and SCom) was very similar. We found differences in the proportions of EO constituents, with the exception of the *J. communis* EO. As mentioned in the Introduction section, *J. communis* is one of the most widely distributed species on the planet [20], and therefore its EO composition has been reported most often. Previous studies on *J. communis* utilized different EO extractions methods [29,30,31,32,33]. It has been demonstrated that supercritical fluid extraction and hydrodistillation methods result in dissimilar leaf EO composition [32].

Our results disprove the working hypothesis, because the EO profile of the samples extracted via the two different methods was very similar within a juniper species. In this study, the main EO constituents of the different extraction methods were α-pinene, sabinene, limonene, β-myrcene. Previous research identified the same composition of EOs obtained by micro-distillation and extraction, and supercritical carbon dioxide extraction from juniper needles [31]. Differences in the EO composition of *J. communis* have been reported following supercritical fluid extraction (SFE) and hydrodistilled EOs [32]. The cited authors found a total of 22 compounds in the EOs from SFE, and only 11 in the hydrodistilled EO [32]. The EO of *J. communis* berries (galbuli) had different qualitative compositions when extracted via hydrodistillation and hexane extraction methods [30]. The concentrations of α-pinene, sabinene, myrcene were higher in the hydrodistilled EO, while some less volatile compounds were present in the extracts, especially in the hexane extract [30].

### 2.3. Insecticidal Action and Repellent Activities of the EOs from the Semi-Commercial Steam Extraction against Rhopalosiphum padi (Bird Cherry—Oat Aphid) and Sitobion avenae (English Grain Aphid)

Aphids are some of the main pests on cereal crops and can significantly reduce yields [34,35]. The interaction effect of species-sex and EO concentration was highly significant on % number (nb) for the repellent action of EO on leaf aphids (Table 5), which suggests that the ideal EO concentration that needs to be used in repellent varies with the combination of the species and the sex of the plant. There are no previous reports concerning repellent and insecticidal action of the EOs of Bulgarian conifers.

#### 2.3.1. Repellent Activity of EOs of *J. oxycedrus*, *J. communis*, *J. pygmaea*, and *J. sibirica*

The insect repellent activity of EOs is commonly used to deter insects. The results of the repellent activity for the EO and constituents against *Rhopalosiphum padi* and *Sitobion avenae* are presented in Table 6. The data of the tested EO of *Rh. padi* showed that at a 5% concentration, the EOs of *J. oxycedrus* (M), *J. sibirica* (M) and *J. pygmaea* (F, M) had a strong repellent activity (Table 6), where the main compound EO in all three species was α-pinene (Table 2). However, at concentrations of 1–2.5%, the EOs *J. communis* (M), *J. sibirica* (F) (1%), *J. oxycedrus* (M), and *J. sibirica* (F) (2.5%) demonstrated a lower repellent action (Table 6). Similar results were obtained by Carroll et al. [36]. The cited authors tested junipers EOs (*J. chinensis, J. communis*) for repellency for *Aedes aegypti*, *Amblyomma americanum* and *Ixodes scapularis* and concluded that the oils were repellent to both species of ticks, but EO of *J. communis* had a minimum effective dosage for repellency [36]. However, in this study, *S. avenae* was more sensitive to the tested EOs at lower concentrations. The three tested concentrations (1%, 2.5% and 5%) of the EOs of *J. communis* (F), *J. sibirica* (F), and *J. oxycedrus* (M) had a strong repellent activity, and α-pinene and sabinene were main EO constituents in the three species. The oils of *J. communis* (M) (2.5% and 5%), *J. pygmaea* (F) (1% and 5%), *J. pygmaea* (M) (2.5% and 5%), and *J. sibirica* (M) (2.5% and 5%) had a lower repellent activity (Table 6).

Overall, in this study, the EOs from *J. communis* (F, M), *J. pygmaea* (F, M) and *J. sibirica* (F, M) were found to have the strongest repellent activity on both aphid species (*Rh. padi* and *S avenae*). The main compounds in the EOs of *J. communis* (F, M), *J. pygmaea* (F, M) and *J. sibirica* (F, M) were α-pinene and sabinene (Table 2). Over 50% of the composition of the *J. communis*, *J. pygmaea* and *J. sibirica* oils consists of α-pinene, sabinene, germacrene D, and δ-cadinene. α-pinene was reported as a repellent in an insect activity study [37]. Obviously, the synergism between the individual compounds in the EOs of *J. communis*, *J. pygmaea* and *J. sibirica* is the reason for their activity. Current thinking is that although some specific compounds may have a repellent effect, in some instances, the interaction between them causes greater bioactivity [38]. The EOs of junipers can be used in lower doses to achieve a repellent effect in agricultural management practices.

#### 2.3.2. Insecticidal Activity of EOs of *J. oxycedrus*, *J. communis*, *J. pygmaea*, and *J. sibirica*

The insecticidal activity of the EOs obtained from the four juniper species (M, F) was established by testing oil efficacy at different concentrations. The effect was calculated using Abbot’s formula [39]. The tested EOs demonstrated a very good insecticidal effect 24 h after the treatment of the aphids (Table 7). The EOs most often act as neurotoxins on the insects, and they affect their physiological processes [40,41]. The EOs’ efficacy was 100% on both aphids (*S. avenae* and *Rh. padi*). An exception was the EO of *J. sibirica* (M) at 2.5% and *J. communis* (F) at 1%, with efficiencies around 90%. After 72 h, the efficiency in all treatments and concentrations was 100% (Table 7). The juniper EOs have mostly been tested for the control of mosquitoes and ticks, and less for the control of agricultural pests. Athanassiou et al. [42] suggested that the simultaneous use of silica gel and EO of *J. oxycedrus* ssp. *oxycedrus* significantly enhanced the activity against *Sitophilus oryzae*. Therefore, the results suggest that the tested EOs could be utilized at their lowest dose (1%) to achieve a very good insecticidal effect [42]. We observed the excellent insecticidal effect of juniper EOs on both species of aphids. The efficacy was 100% after 24 h of the EO applications. These results are not accidental, because α-pinene, sabinene, limonene, β-myrcene are the main constituents of monoterpenes in juniper species and they have been reported to have insecticidal activity [43,44,45]. Monoterpenes and sesquiterpenes (α-pinene, terpineol, 1,8-cineole, p-cymene, limonene, α-terpinene, thymol, carvacrol) were found to have a high fumigant activity against *Musca domestica*, *Tribolium confusum* and *Sitophilus oryzae* [43,45].

### 2.4. Antifungal Activity of Juniper EOs on Plant Pathogenic Fungi

Screening of the antifungal activity of the four juniper species EOs (1 µL mL^−1^) on the studied plant pathogens showed a varying positive effect (Table 8). The most substantial inhibitory effect on the radial mycelial growth was found against *C. pauciseptatum* and *Fusarium* sp. Significantly different inhibitory effects (*p* < 0.05) on the mycelial growth of *Fusarium* sp. (36.6% and 34.5%) were observed after the application of the EOs from *J. sibirica* (F) and *J. pygmaea* (M). Regarding the other tested EOs, the closest values to that already mentioned were obtained from the EOs of *J. oxycedrus* (M) and *J. communis* (M), and the differences in their antifungal activity were small. The largest difference in the antifungal activity of EOs obtained from M and F juniper species was between EOs from *J. pygmaea* (M) and *J. pygmaea* (F) (16.9%). The weakest inhibitory effect was found for the *J. communis* (F) EO against *Fusarium* spp. (Table 8).

The highest antifungal efficacy against *C. pauciseptatum* was observed in *J. oxycedrus* (M), *J. pygmaea* (M), *J. sibirica* (F), *J. sibirica* (M) and *J. pygmaea* (F). The inhibition coefficient varied from 29.8% to 24.7%. A statistically similar inhibitory effect was found in *J. oxycedrus* (M) and *J. pygmaea* (M), as well as in *J. pygmaea* (F) and *J. sibirica* (M). A significantly different inhibitory effect was found among *J. oxycedrus* (M), *J. pygmaea* (F), *J. communis* (F) and *J. communis* (M). Statistically significant difference (10.96%) was established in the percent inhibition of the EOs from sexually different species (*J. communis* F and *J. communis* M) on the mycelial growth of *C. pauciseptatum*. The inhibition coefficient of *J. pygmaea* (M) on the mycelial growth of *R. solani* and *C. pauciseptatum* was the highest and significant compared to the other studied EOs.

*Juniperus pygmaea* (M) EO showed a strong inhibitory efficacy against four of the studied pathogens (*Fusarium* sp., *R. solani*, *C. pauciseptatum* and *Colletotrichum* sp.), while *J. sibirica* (F) showed a similar effect against *Fusarium* sp. and *C. pauciseptatum* (Table 8). As shown in Table 2, the concentrations of sabinene (25.06), terpinen-4-ol (6.01) and α-pinene (19.64) were higher in *J. pygmaea* (M), and these compounds probably interact with each other. *Botrytis cinerea* was the most resistant species against the used juniper EOs. None of the tested EOs showed a significant inhibitory effect on mycelial growth of *B. cinerea*.

The available information on the biological activity of the tested juniper EOs against phytopathogenic fungi is scarce. Zabka et al. [46] reported relatively low inhibitory activity of EO from *J. communis* L. on *Alternaria alternata*, *Cladosporium cladosporioides* and *Aspergilus niger*, 11.8%, 31.0% and 1.8%, respectively, at 1 μL/mL. According to these authors, the high antifungal effect of *Origanum vulgare, Thymus vulgaris* and *Pimenta racemosa* EO of the 20 studied types of oils was due to the high content of the phenolic monoterpenes thymol, carvacrol or eugenol. Gleń-Karolczyk and Boligłowa [47] reported a strong inhibitory effect of *J. communis* EO, ranging from 87.1% at 0.1 mm^3^ cm^−3^ to 93.1% at 1 mm^3^ cm^−3^ on *R. solani* and from 42% at 0.1 mm^3^ cm^−3^ to 85.4% at 1 mm^3^ cm^−3^ on *C. destructans* isolated from horseradish seedlings. Analyzing EOs from green and ripe berries of *J. communis*, an antifungal effect was found, manifested against *Sclerotium rolfsii* [48]. Based on their own results and on those obtained by Jing et al. [49], the latter authors suggested that sesquiterpenes were the phytochemical components that show a toxic effect on fungal pathogens. It can be seen that EOs mainly from *J. communis* were tested to establish the antifungal effect against phytopathogens.

In the present study, the most significant inhibition of the radial mycelial growth of the studied phytopathogens was demonstrated by the EOs of *J. sibirica* (F) (36.6%) and *J. pygmaea* (M) (34.5%) on *Fusarium* spp., by the EO of *J. oxycedrus* (M, 29.8%) and *J. pygmaea* (M, 29.0%) on *C. pauciseptatum*, as well as by the EO of *J. pygmaea* (M, 31.97%) on *R. solani*.

In most cases, there were statistically significant differences between the means of the inhibition coefficient of the juniper EO from M and F plants against the tested pathogens (Table 8). This was probably due to the different chemical composition of the EOs obtained from M and F plants. An exception was found in EO from *J. communis* against *C. pauciseptatum* and *J. sibirica* against *R. solani* and *C. pauciseptatum*, in which, under the conditions of the present experiment, the differences between the inhibitory effect of EO obtained from M and F juniper plants were not significant.

Overall, we cannot find a clear pattern between the chemical composition of EOs of *Juniperus* species and their inhibitory effect. *J. pygmaea* and *J. sibirica* are characterized with a high content of sabinene (Table 2). In the studied species, the predominant component of EOs was the class monoterpenes, especially α-pinene. In order to determine exactly which compounds exhibit antifungal efficacy, these compounds should be tested individually. As indicated by Tripathietii et al. [50] and Sharma and Tripathi [51], the fungicidal activity of EO is often the result of the synergistic activity of their compounds.

The results from this study on the inhibitory effect of EOs from four species of juniper, M and F plants (1 µL/mL) on phytopathogens and polyphages, causing diseases both during vegetation and the storage of plant products, are encouraging and call for further research.

### 2.5. Antimicrobial Activity

The antimicrobial activity of the F and M *Juniperus* EOs (*J. communis*, *J. oxycedrus*, *J. pygmaea*, *J. sibirica*) obtained through the commercial steam extraction method was evaluated in this study. Among the combinations of the levels of the two factors (sex, species), the highest antimicrobial activity was observed in *J. communis* (M) and *J. sibirica* (M) on S. *pneumoniae*; *J. sibirica* (F), *J. communis* (M), *J. pygmaea* (M) on *C. perfringens*; *J. pygmaea* (M, F) on *L. monocytogenes*; *J. sibirica* (F) on *E. faecalis*; *J. sibirica* (F) on *S. enterica*; *J. pygmaea* (F), *J. sibirica* (M, F) on *H. influenzae*; *J. sibirica* (F) on *P. aeruginosa*; and *J. sibirica* (F) on *Y. enterocolitica* (Table 9 and Table 10). High antimicrobial activity was observed for the EO of *J. sibirica* (M, F) on the seven tested bacterial species (*S. pneumoniae*, *C. perfringens*, *E. faecalis*, *S. enterica*, *H. influenzae*, *P. aeruginosa*, and *Y. enterocolitica*), followed by the EOs of *J. communis* (M) and *J. pygmaea* (M, F). Similar antimicrobial activity was observed for the EOs of *J. communis* on Gram-positive and Gram-negative bacterial species and yeasts [14,52], while Glišič et al. [53] found higher antimicrobial activity for *Juniperus* EOs compared with that of commercial antibiotics. Overall, the results of this study confirm our working hypothesis.

### 2.6. Antioxidant Activity

**Figure 1:**
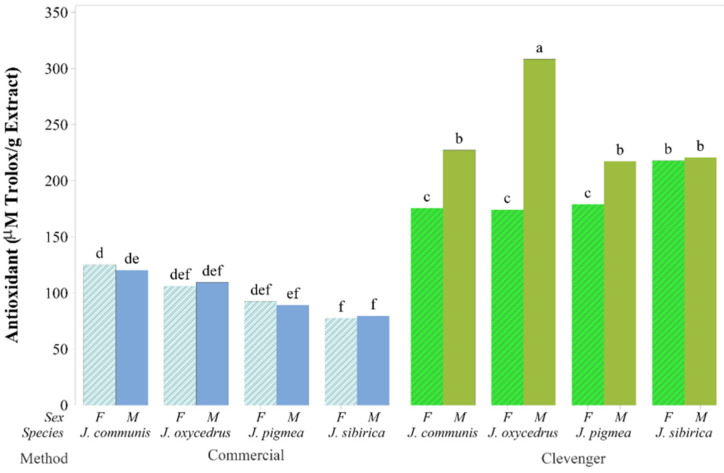
Bar graph of mean antioxidant capacity in μmol Trolox g^−1^ obtained from the 16 combinations of the extraction method, species and sex. Means sharing the same letter (at the top of the bars) are not significantly different.

Essential oils, including those from some of the juniper species, have been utilized in the pharmaceutical, cosmetic and food industries [5]. The antioxidant capacity (ORAC) of *J. communis*, *J. oxycedrus*, *J. pygmaea*, and *J. sibirica* EOs obtained by different extraction methods were evaluated in this study. The ORAC values of Clevenger method EOs were higher than that of semi-commercial method EOs (Figure 1). The results on the antioxidant capacity confirm our working hypothesis that the EOs extracted via different methods would have dissimilar bioactivity. Overall, the values of monoterpenes in the tested EOs obtained by the Clevenger method were higher than the concentration of monoterpenes in the EOs obtained using the semi-commercial method, and α-pinene was the predominant constituent. Reports on the antioxidant capacity of junipers EOs can be found in the literature [7,8,9,10,14,23,24]. It is well known that the qualitative and quantitative profiles of junipers can be influenced by a number of factors, including genetic, environmental, and post-harvest processing such as drying and extraction. The higher antioxidant capacity of the galbuli EO from *J. sibirica* and *J. communis* were reported by Zheljazkov et al. [14], where α-pinene was the dominant compound in the EOs. Due to the high level of γ-terpinene, Emami et al. [54] reported the strong antioxidant capacity of the leaves *J. communis* subsp. *hemisphaerica* [54]. In this study, the highest ORAC value was obtained from EOs of *J. oxycedrus* (M) (Figure 1), where α-pinene and limonene were the dominant compounds (Table 2). Current thinking is that the antioxidant capacity in EOs may depend strongly on the interaction between several compounds. The demonstrated high antioxidant capacity of some of the juniper oils opens the door for their wider utilization in various products.

## 3. Materials and Methods

### 3.1. Plant Material

In this study, plant materials of *Juniperus communis* L., *J. oxycedrus* L., *J. pygmaea* C. Koch., and *J. sibirica* Burgsd. were used. Samples of the four species were collected from natural populations as follows: (а) *J. communis* (F, M) samples were collected above the village of Markovo in the Rhodope Mountains, 42°02′35.2″ N; 024°42′05.6″ E, at 587 masl; (b) *J. oxycedrus* (F, M) samples were also collected above the village of Markovo in the Rhodope Mountains, 42°02′32.6″ N; 024°42′05.7″ E, at 613 masl; (c) *J. pygmaea* (F, M) samples were collected above the village of Dobrostan in the Rhodope Mountains, 41°54′12.4″ N; 024°55′02.05″ E, at 1314 masl; and (d) *J. sibirica* (F, M) samples were collected in the Trojan mountain pass (Beklemeto) of Stara Planina (The Balkans) Mountains, 42°46′16.4″ N; 024°36′43.6″ E, at 1485 masl. Voucher specimens of *J. communis, J. oxycedrus, J. pygmaea,* and *J. sibirica* (small branches with needles) were deposited at the Herbarium of the Agricultural University, Plovdiv, Bulgaria (SOA) [55].

### 3.2. Preparation of Juniperus Samples for the Extraction of the Essential Oil (EO)

The samples of the four Juniper species were collected in June and were placed in a shady area at a temperature of below 35 °C for air-drying.

### 3.3. Hydrodistillation Extraction of EO

The Clevenger-type (ClevA) hydrodistillation extraction of EO was performed according to the British Pharmacopoeia [56] by using 800 mL of water in 2 L Clevenger-type hydrodistillation units. The plant tissue samples consisted of 100 g of air-dried leaves in 2 L flasks for the ClevA distillation. All samples were distilled for 2 h in two replicates.

### 3.4. Steam Distillation Semi-Commercial Extraction (SCom) of EO

The SCom steam distillation was conducted in a semi-commercial 100 L steam distillation units using 20 kg of leaves and small twigs, steam distilled for 3 h, the usual steam distillation time used by industry for junipers. The resulting EO from the above extractions was collected, separated from the remaining water, and kept in a freezer until the gas chromatography (GC)-mass spectroscopy (MS) analyses were performed. The EO was measured both by volume and by weight.

### 3.5. Gas Chromatography (GC)—Mass Spectroscopy (MS) Analyses of the Essential Oils (EO)

The chemical profile of the four *Juniperus* EOs in three replications was determined by GC-FID and GC/MS techniques, according to the methods described previously [14]. Identification of the components present in the EO samples was performed by comparing the mass spectra of components in the EOs with those from the National Institute of Standards and Technology (NIST 08) and Adams mass spectra libraries [57], by AMDIS (Automated Mass spectral Deconvolution and Identification System) and by comparing the literature and estimated Kovat′s (retention) indices that were determined using mixtures of homologous series of normal alkanes from C8 to C40 in hexane, under the same conditions mentioned above. The percentage ratio of EO components was computed using the normalization method of the GC/FID peak areas (Supplementary Materials).

### 3.6. Testing the Repellent and Insecticidal Action of EOs Obtained via Semi-Commercial Steam Extraction against Rhopalosiphum padi (Bird Cherry—Oat Aphid) and Sitobion avenae (English Grain Aphid)

#### 3.6.1. Colonization of Rhopalosiphum padi and *Sitobion avenae*

The experiment was conducted at the entomology laboratory of the Institute of Agriculture in Karnobat, Bulgaria. Wingless female aphids of the above species were used in the experiment. The *Rhopalosiphum padi* and *Sitobion avenae* aphids were collected from the barley fields in the area of Karnobat, Bulgaria (42°38′54.51″ N, 27°21′60.56″ E). The aphids were kept on *Hordeum vulgare* Jess. subsp. *distichum* L., var. *erectum*, cv. Obzor plants which were grown in containers under controlled environment conditions. After the young barley plants reached the 3rd leaf stage, the aphids were introduced and infested the experimental plants. The experimental conditions included a temperature of 23–24 °C, 65% RH, and a light:dark (L:D) cycle of 8:16 h.

#### 3.6.2. Repellency Tests

Each of the EOs extracted by SCom steam distillation of *J. communis* (F, M), *J. oxycedrus* (M), *J. pygmaea* (F, M), and *J. sibirica* (F, M) were tested for their repellent activity against *Rh. padi* and *S. avenae.* The repellence of the EO was assessed by using the Petri dish assay [58]. The seven EOs were diluted with an aqueous solution with an emulsifier, 0.1% polysorbate 80. Two microliters of each EO of the target species with 0%, 1%, 2.5%, and 5% concentrations in three replicates were used, according to the methods described previously [10]. One treated leaf and one non-treated leaf plus 10 leafless aphids were introduced into each Petri dish. After that, the Petri dishes were covered with cheesecloth (44 g/m^2^). The repellent effect was observed and recorded after 24 h.

#### 3.6.3. Testing the Insecticidal Action of the Essential Oils (EO) from the SCom Steam Distillation against *Rhopalosiphum padi* and *Sitobion avenae*

The insecticidal activity of EOs was determined following a method described by Konstantopoulou et al. [59]. Each of the seven EOs was tested on the adult wingless forms of two aphid species at a concentration of 0%, 1%, 2.5%, and 5% in three replicates. The insecticidal actions of the EO on pests were evaluated on leaves of *H. vulgare* subsp. *distichum* var. *erectum*, cv. Obzor. The EOs were diluted in aqueous solution with an emulsifier of 0.1% polysorbate 80. The control (0%) was treated with a 0.1% aqueous solution of polysorbate 80. Two microliters of the solution (0%, 1%, 2.5%, and 5%) were applied directly on barley leaves with the aphid colonies. After the treatments, the leaves were dried on filter paper and transferred to Petri dishes [59]. The Petri dishes were covered with cheesecloth (44 g/m^2^). The effect of the application (knock-down or mortality) was observed after 24 and 72 h. The results (knock-down or mortality) were compared with the controls. The effect of the application of EOs at different concentrations was calculated using Abbott’s formula [38]:

E = (x − y)/x × 100

where E is aphid mortality; x is the percentage of live aphids in the control; and y is % of live aphids in the treatment with the EOs.

### 3.7. Antifungal Activity of the Juniper EO on Plant Pathogenic Fungi

#### 3.7.1. Fungal Plant Pathogenic Strains

Five plant pathogenic fungal strains were obtained from a culture collection maintained in the Department of Phytopathology, Agricultural University, Plovdiv, Bulgaria. *Fusarium* sp. (fusarium dry rot) and *Rhizoctonia solani* (stem canker and black scurf) were isolated from stored potato tubers (*Solanum tuberosum* L.), *Botrytis cinerea* (grey mould) strain was isolated from infected stored tomato fruits (*Lycopersicon esculentum* Mill). The strain of a *Colletotrichum* sp. was isolated from anthracnose of banana fruit (*Musa* sp.), whereas *Cylindrocarpon pauciseptatum* strain was obtained from black foot disease of grapevine (*Vitis vinifera* L.). All strains were identified using cultural and morphological characteristics, as well as pathogenicity test. The *Cylindrocarpon pauciseptatum* was confirmed by DNA sequences [60]. Strains were preserved on potato dextrose agar (PDA) at 4 °C.

#### 3.7.2. Agar Dilution Method (Antifungal Activity of Juniper EOs)

Preliminary study of inhibitory effect of seven juniper essential oils (*J. oxycedrus* L. M, *J. communis* L. M and F, *J. pygmaea* C. Koch., M and F, and *J. sibirica* Burgsd. M and F) on the mycelial radial growth of plant pathogenic fungi were tested by the agar dilution method [46]. The essential oils were diluted in PDA at 1 µL mL^−1^ concentration. Discs (5 mm/d) were cut out from the periphery of a 10 day-old culture of tested fungi and aseptically placed in the prepared Petri dishes (9 cm/d) with PDA and EO. Pure PDA medium (without essential oil) was used as the control. The inoculated Petri dishes were placed for incubation at 22 °C for 10 days. The percent inhibition of the radial mycelial growth of the tested fungal pathogens was calculated using the formula:

(DC − DT)/DC × 100%

where DC is the diameter of the colony of the control, and DT is the diameter of the colony of the treatment. The experiment was conducted in four replications.

### 3.8. Antioxidant Activity

The antioxidant activity of EOs of *J. communis*, *J. oxycedrus*, *J. pygmaea* and *J. sibirica* (Clevenger; Commercial) were analyzed at the University of Nebraska-Lincoln, Small Molecule Analysis Laboratory. The oxygen radical absorbance capacity (ORAC oil) was detected according to Huang et al. [61,62]. Briefly, Trolox (6-hydroxy-2,5,7,8-tetramethylchroman-2-carboxylic acid), a polar derivative of vitamin E, was used as a standard, and the results were reported as µmole Trolox g^−1^. All samples of EOs were prepared by mixing 10 ± 1 mg oil with 1 mL of water and acetone (1:1) with 7% methyl-β-cyclodextrins (*w*:*v*). The activity was started in a 96-well plate by first transferring 25 μL of 74 mM phosphate buffer, with pH 7.4, to each well. Thereafter the EO samples (25 µL) or Trolox (25 μL) were added at concentrations of 0.2, 0.4, 3.3, 6.5, 10, 13, 25, and 50 μg/mL, followed by 150 μL of fluorescein (8.16 × 10^−5^ mM). Each sample was incubated at 37 °C for 10 min, with 3 min of alternating shaking. The 153 mM 2,2′-azobis (2-amidinopropane) hydrochloride (25 μL) was used for reaction activation. The standards and tested EO were monitored with a BMG Labtech FLUOstar Optima microplate reader (Durham, NC, USA). We measured fluorescence every 1.5 min at an excitation and emission wavelength of 485 and 520 nm, respectively, until the decreasing fluorescence values plateaued. The area under the curve was calculated. The EOs of the four junipers were analyzed in triplicate. The averages of the tested EOs in triplicate were used for the statistical analysis.

### 3.9. Antimicrobial Activity

#### 3.9.1. Microorganisms

The four Gram-negative bacteria (*Salmonella enterica* susp. *enterica* CCM 3807, *Haemophilus influenzae* CCM 4456, *Pseudomonas aeruginosa* CCM 1959, and *Yersinia enterocolitica* CCM CCM 5671), four Gram-positive bacteria (*Clostridium perfringens* CCM 4435, *Enterococcus faecalis* CCM 4224, *Listeria monocytogenes* CCM 4699 and *Streptococcus pneumoniae* CCM 4501), were used for the antimicrobial activity testing. The microorganisms were used from the Czech Collection of Microorganisms, Brno, Czech Republic. The cultures were incubated in Mueller–Hinton broth (MHB, Oxoid, Basingstoke, UK) at 37 °C overnight.

#### 3.9.2. Disc Diffusion Method

One hundred microliters of bacterial suspension after incubation were spread on the Mueller–Hinton agar (MHA, Oxoid) for the agar disc diffusion method. The filter paper discs (6 mm diameter) were infused with 15 µL of the EO, tested, and placed on the inoculated MHA. The MHA was kept at 4 °C for 2 h and then at 37 °C for 24 h aerobically. For yeasts, 100 µL of the microbial suspension was spread onto Sabouraud agar (Oxoid) and cultivated at 37 °C for 24 h. After the incubation period, the diameter of the inhibition zones was measured (mm). The growth inhibition was compared with standard drugs. Tests were performed in three replications.

### 3.10. Statistical Analyses

#### 3.10.1. Statistical Methods for Constituents, Antimicrobial Activities, and Antioxidant Capacity

The effect of juniper species (4 levels: *J. communis*, *J. oxycedrus*, *J. pygmaea*, and *J. sibirica*) and sex (2 levels: F and M) on (1) the concentrations of 21 constituents (α-pinene, camphene, sabinene, β-pinene, β-myrcene, α-terpinene, p-cymene, limonene, γ-terpinene, α-terpinolene, terpinen-4-ol, bornyl acetate, β-elemene, β-caryophyllene, α-humulene, germacrene D, γ-cadinene, δ-cadinene, δ-cadinol, tau.-cadinol, and α-cadinol), and (2) eight antimicrobial activities (*S. pneumoniae*, *C. perfringens*, *L. monocytogenes*, *E. faecalis*, *S. enterica*, *H. influenzae*, *P. aeruginosa*, and *Y. enterocolitica*) where the extraction method was commercial was determined using a 4 × 2 factorial design. For antioxidant capacity response, in addition to the above two factors, a third factor, namely extraction method (2 levels: ClevA and SCom), was added, which made the design a 4 × 2 × 2 factorial.

#### 3.10.2. Statistical Methods for Aphids

The effect of the combinations of juniper species and sex (7 levels: *J. communis*-F, *J. communis*-M, *J. oxycedrus*-M, *J. pygmaea*-F, *J. pygmaea*-M, *J. sibirica*-F, and *J. sibirica*-M) and the concentration of their EOs (4 levels: 0%, 1%, 2.5%, and 5%) on nb/leaf of *Sitobion avenae* and *Rhopalosiphum padi* for repellent action was determined using a 7 × 4 factorial design with 3 replications. However, for the insecticidal action, only three concentrations (1%, 2.5%, and 5%) were used for each of the seven species by sex combinations. Additionally, the antifungal activity effect of juniper species and sex (7 levels) on plant pathogens was determined using a completely randomized design (a single factor with 7 levels).

The analysis was completed using the GLM procedure of SAS [63], and the validity of model assumptions (normal distribution and constant variance of the error terms) was verified by examining the residuals as described in Montgomery [64]. Since the two-way interaction effect for all constituents, antimicrobial activities, and aphids and the three-way interaction effect for antioxidant capacity were highly significant, a multiple means comparison of the corresponding treatment combinations was completed and letter groupings generated using the least squares means at the 1% level of significance to protect the Type I experiment-wise error rate from overinflation.

## 4. Conclusions

Generally, the EOs of *J. communis*, *J. pygmaea*, *J. sibirica*, and *J. oxycedrus* had similar chemical profiles between the two extraction methods (ClevA, SCom) within a species, but they had a different antioxidant capacity. The EOs obtained via ClevA extraction showed greater antioxidant capacity within a species compared with those from SCom extraction. Overall, the EOs among the two factors (sex, species) had different antimicrobial activity. All of the tested EOs had significant repellent and insecticidal activity against the two aphid species *Rhopalosiphum padi* (bird cherry-oat aphid) and *Sitobion avenae* (English grain aphid) at concentrations of the EO in the solution at 1%, 2.5%, and 5%. Most of juniper EOs were effective against the tested pathogens, *Fusarium* spp., *Cylindrocarpon pauciseptatum Colletotrichum* spp. and *Rhizoctonia solani*, especially *J. pygmaea* (M) and *J. sibirica* (F). These EOs show promise as alternatives to conventional synthetic pesticides and could be used in the development of biopesticide products.

## Figures and Tables

**Table 1:** Essential oil (EO) content of *Juniperus oxycedrus*, *J. communis*, *J. pygmaea*, and *J. sibirica* as a function of the sex of the tree and the extraction method.

Species	Sex	EO Yield (%)—Clevenger	EO Yield (%)—Commercial
*J. oxycedrus*	M	0.08	0.07
*J. oxycedrus*	F	0.20	0.05
*J. sibirica*	M	0.36	0.30
*J. sibirica*	F	0.31	0.32
*J. communis*	M	0.32	0.24
*J. communis*	F	1.63	0.61
*J. pygmaea*	M	0.84	0.51
*J. pygmaea*	F	1.60	0.35

M—male; F—female.

**Table 2:** Mean concentration (%) of α-pinene, camphene, sabinene, β-pinene, β-myrcene, α-terpinene, and p-cymene (extracted using the commercial steam extraction method) obtained from the eight combinations of species and sex.

Species	Sex	α-Pinene	Camphene	Sabinene	β-Pinene	β-Myrcene	α-Terpinene	p-Cymene
*J. communis*	F	34.91 a *	0.32 a	9.77 d	1.78 d	4.66 a	0.87 b	0.96 cd
*J. communis*	M	26.81 c	0.31 b	9.28 d	1.69 d	4.43 b	0.83 b	0.91 d
*J. oxycedrus*	F	32.19 b	0.25 c	0.97 e	3.97 b	3.21 c	0.34 d	0.28 g
*J. oxycedrus*	M	26.74 c	0.27 c	1.58 e	0.45 f	0.40 f	0.13 e	1.37 a
*J. pygmaea*	F	21.88 d	0.18 e	15.12 b	4.54 a	1.06 e	0.86 b	0.99 c
*J. pygmaea*	M	19.64 e	0.2 d	25.06 a	2.31 c	1.55 d	1.57 a	1.28 b
*J. sibirica*	F	14.86 f	0.17 e	13.67 c	0.69 e	1.06 e	0.62 c	0.51 f
J. sibirica	M	20.15 e	0.15 f	15.38 b	0.59 ef	1.19 e	0.15 e	0.63 e

* Within each constituent, means sharing the same letter are not significantly different.

**Table 3:** Mean concentration (%) of limonene, γ-terpinene, α-terpinolene, terpinen-4-ol, bornyl acetate, β-elemene, and β-caryophyllene (extracted using the commercial steam extraction method) obtained from the eight combinations of species and sex.

Species	Sex	Limonene	γ-Terpinene	α-Terpinolene	Terpinen-4-ol	Bornyl Acetate	β-Elemene	β-Caryophyllene
*J. communis*	F	2.14 d *	1.47 c	1.13 d	5.15 b	0.66 a	0.33 f	2.42 c
*J. communis*	M	7.22 a	1.52 bc	0.96 e	3.98 c	0.32 d	1.16 c	3.69 a
*J. oxycedrus*	F	3.12 c	0.51 f	0.49 f	0.32 f	0.66 a	0.90 e	2.11 d
*J. oxycedrus*	M	5.00 b	0.53 f	0.52 f	0.34 f	0.69 a	0.95 de	1.02 f
*J. pygmaea*	F	1.47 e	1.57 b	1.07 d	3.09 e	0.44 b	4.77 b	3.19 b
*J. pygmaea*	M	2.15 d	2.76 a	1.92 a	6.01 a	0.38 c	1.11 cd	0.93 f
*J. sibirica*	F	1.24 e	1.27 d	1.21 c	3.65 d	0.34 d	6.80 a	1.05 f
*J. sibirica*	M	1.27 e	1.15 e	1.37 b	3.52 d	0.44 b	1.05 cde	1.24 e

* Within each constituent, means sharing the same letter are not significantly different.

**Table 4:** Mean concentration (%) of α-humulene, germacrene D, γ-cadinene, δ-cadinene, δ-cadinol, tau-cadinol, and α-cadinol (extracted using the commercial steam extraction method) obtained from the eight combinations of species and sex.

Species	Sex	α-Humulene	Germacrene D	γ-Cadinene	δ-Cadinene	δ-Cadinol	Tau.-Cadinol	α-Cadinol
*J. communis*	F	1.34 d *	6.53 e	1.57 c	4.36 d	0.97 b	0.49 f	0.74 c
*J. communis*	M	2.73 b	6.87 e	1.61 c	6.33 ab	1.06 a	0.74 d	0.94 c
*J. oxycedrus*	F	1.47 c	17.75 a	4.03 a	4.34 d	0.80 c	0.82 c	0.65 c
*J. oxycedrus*	M	0.35 g	3.67 f	3.97 a	2.98 e	0.97 b	1.57 a	0.79 c
*J. pygmaea*	F	2.98 a	11.42 c	3.23 b	5.17 c	0.25 e	0.21 g	1.63 b
*J. pygmaea*	M	0.53 f	2.51 g	1.44 cd	2.10 f	0.50 d	0.63 e	2.80 a
*J. sibirica*	F	1.37 cd	9.04 d	1.32 d	5.98 b	0.95 b	1.17 b	2.94 a
*J. sibirica*	M	1.16 e	13.08 b	0.51 e	6.66 a	0.76 c	0.59 e	1.48 b

* Within each constituent, means sharing the same letter are not significantly different.

**Table 5:** ANOVA *p*-values that show the significance of the main effects and the interaction effect of species-sex and concentration (Conc) on nb./leaf of *Rhopalosiphum padi* and nb./leaf of *Sitobion avenae* for repellent action.

Source of Variation	nb./Leaf of *Rh. padi*	nb./Leaf of *S.avenae*
SpeciesSex	0.301	0.001
Conc	0.001	0.001
SpeciesSex * Conc	**0.001**	**0.001**

* Significant effects that require a multiple means comparison are shown in bold.

**Table 6:** Mean *Rhopalosiphum padi* and *Sitobion avenae* for the repellent action obtained from the 28 combinations of species-sex and EO concentration (Conc).

Species-Sex	Conc (%)	nb./Leaf *Rh. padi*	nb./Leaf *S. avenae*	Species-Sex	Conc	nb./Leaf *Rh. padi*	nb./Leaf *S. avenae*
*J.communis*-F	0	20.3 abc *	31.1 b	*J. pygmaea*-M	0	56.3 a	23.3 b
*J. communis*-F	1	8.3 abc	0.0 d	*J. pygmaea*-M	1	8.3 abc	3.3 d
*J. communis*-F	2.5	11.6 abc	0.0 d	*J.pygmaea*-M	2.5	6.5 abc	0.0 d
*J. communis*-F	5	6.5 abc	0.0 d	*J. pygmaea*-M	5	0.0 c	0.0 d
*J. communis*-M	0	18.9 abc	33.3 b	*J. sibirica*-F	0	47.2 ab	62.2 a
*J. communis*-M	1	1.1 c	6.7 cd	*J. sibirica*-F	1	1.1 c	0.0 d
*J. communis*-M	2.5	19.1 abc	0.0 d	*J. sibirica*-F	2.5	1.1 c	0.0 d
*J. communis*-M	5	13.0 abc	0.0 d	*J. sibirica*-F	5	13.0 abc	0.0 d
*J. oxycedrus*-M	0	22.1 abc	20.0 bc	*J. sibirica*-M	0	43.8 ab	53.3 a
*J. oxycedrus*-M	1	13.0 abc	0.0 d	*J. sibirica*-M	1	2.2 bc	3.3 d
*J. oxycedrus*-M	2.5	1.1 c	0.0 d	*J. sibirica*-M	2.5	24.0 abc	0.0 d
*J. oxycedrus*-M	5	0.0 c	0.0 d	*J. sibirica*-M	5	0.0 c	0.0 d
*J. pygmaea*-F	0	26.4 abc	24.5 b				
*J. pygmaea*-F	1	25.9 abc	0.0 d				
*J. pygmaea*-F	2.5	45.2 ab	26.7 b				
*J. pygmaea*-F	5	0.0 c	0.0 d				

* Among the 28 means of each of *Rhopalosiphum padi* and *Sitobion avenae*, means sharing the same letter are not significantly different.

**Table 7:** The insecticidal effect of semi-commercial extraction EOs of *J. pygmaea* (M, F), *J. oxycedrus* (M, F), *J. sibirica* (M, F) and *J. communis* (M, F) on two aphids (*Rhopalosiphum padi, Sitobion avenae*).

Species-Sex	EO Concentrations (%)	*Rh. padi*		*S. avenae*	
		after 24 h	after 72 h	after 24 h	after 72 h
*J. pygmaea*-M	1	100	100	100	100
*J. pygmaea*-M	2.5	100	100	100	100
*J. pygmaea*-M	5	95.1	100	100	100
*J. oxycedrus*-M	1	100	100	100	100
*J. oxycedrus*-M	2.5	100	100	100	100
*J. oxycedrus*-M	5	100	100	100	100
*J. sibirica*-F	1	100	100	100	100
*J. sibirica*-F	2.5	100	100	100	100
*J. sibirica*-F	5	100	100	100	100
*J. pygmaea*-F	1	100	100	100	100
*J. pygmaea*-F	2.5	100	100	100	100
*J. pygmaea*-F	5	100	100	100	100
*J. communis*-M	1	97.1	100	100	100
*J. communis*-M	2.5	100	100	100	100
*J. communis*-M	5	100	100	100	100
*J. sibirica*-M	1	92.7	100	100	100
*J. sibirica*-M	2.5	100	100	96.9	100
*J. sibirica*-M	5	100	100	100	100
*J. communis*-F	1	100	100	92.5	100
*J. communis*-F	2.5	100	100	100	100
*J. communis*-F	5	100	100	100	100

**Table 8:** Inhibitory effect (%) of juniper essential oils on plant pathogenic fungi at concentration of 1 µL/mL.

Species	Sex	*Fusarium* sp.	*B. cinerea*	*Colletotrichum* sp.	*R. solani*	*C. pauciseptatum*
*J.oxycedrus*	M	19.75 d *	0.00 b	0.00 d	13.85 c	29.77 a
*J.communis*	F	15.50 f	5.75 a	11.95 b	16.25 b	9.55 f
*J.communis*	M	19.00 de	0.35 b	11.40 bc	5.60 e	20.51 e
*J. pygmaea*	F	17.63 e	0.00 b	12.12 b	11.10 d	24.70 d
*J. pygmaea*	M	34.53 b	0.00 b	17.05 a	31.97 a	29.00 ab
*J. sibirica*	F	36.63 a	0.00 b	0.00 d	11.10 d	27.44 bc
*J. sibirica*	M	28.90 c	0.00 b	10.02 c	12.10 d	26.20 cd

* Within each column, means sharing the same letter are not significantly different.

**Table 9:** Mean antimicrobial activity (inhibition zones in mm) of *S. pneumoniae*, *C. perfringens*, *L. monocytogenes*, and *E. faecalis* obtained from the eight combinations of species and sex.

Species	Sex	*S. pneumoniae*	*C. perfringens*	*L. monocytogenes*	*E. faecalis*
*J. communis*	F	5.00 de *	3.67 c	4.33 c	5.33 c
*J. communis*	M	11.67 a	7.33 ab	5.33 bc	2.33 d
*J. oxycedrus*	F	3.33 e	3.33 c	5.33 bc	3.33 d
*J. oxycedrus*	M	4.00 de	3.33 c	5.33 bc	3.33 d
*J. pygmaea*	F	6.00 cd	6.00 b	10.67 a	3.33 d
*J. pygmaea*	M	9.00 b	7.33 ab	9.33 a	8.33 b
*J. sibirica*	F	7.67 bc	8.67 a	10.33 a	10.67 a
*J. sibirica*	M	11.67 a	4.33 c	6.33 b	2.33 d

* Within each antimicrobial activity, means sharing the same letter are not significantly different.

**Table 10:** Mean antimicrobial activity (inhibition zones in mm) of *S. enterica*, *H. influenzae*, *P. aeruginosa*, *Y. enterocolitica*, and *S. pneumoniae* obtained from the eight combinations of species and sex.

Species	Sex	*S. enterica*	*H. influenzae*	*P. aeruginosa*	*Y. enterocolitica*
*J. communis*	F	4.33 bc *	3.67 d	4.33 b	3.67 b
*J. communis*	M	3.33 c	7.33 b	5.33 b	5.33 b
*J. oxycedrus*	F	4.33 bc	5.67 c	4.67 b	5.33 b
*J. oxycedrus*	M	5.33 b	5.00 cd	4.67 b	5.00 b
*J. pygmaea*	F	5.33 b	10.67 a	5.33 b	5.33 b
*J. pygmaea*	M	5.33 b	7.33 b	5.67 b	5.00 b
*J. sibirica*	F	8.33 a	10.33 a	9.67 a	8.67 a
*J. sibirica*	M	3.67 c	11.00 a	5.33 b	4.67 b

* Within each antimicrobial activity, means sharing the same letter are not significantly different.

## Data Availability

Data of the compounds are available from the authors.

## Supplementary Materials

The following are available online, Table S1: Constituents and concentrations of *J. communis* extracted using the Clevenger and Commercial extraction methods; Table S2: Constituents and concentrations of *J. sibirica* extracted using the Clevenger and commercial extraction methods; Table S3: Constituents and concentrations of *J. pygmaea* extracted using the Clevenger and commercial extraction methods; Table S4: Constituents and concentrations of *J. oxycedrus* extracted using the Clevenger and Commercial extraction methods; Table S5: Main class of compounds in the essential oil (EO) of *J. oxycedrus*, *J. communis*, *J. pygmaea*, *J. sibirica*.

## Sample Availability

Data of the compounds are available from the authors.

## Abbreviations

MH—monoterpenes hydrocarbons; OM—oxygenated monoterpenes; PhM—phenolic monoterpenes; BOM—bicyclic oxygenated monoterpenes; SH—sesquiterpenes hydrocarbons; OS—oxygenated sesquiterpenes; D—diterpenes; OD—oxygenated diterpenes; TOS—tricyclic oxygenated sesquiterpenes; BSH—bicyclic sesquiterpenes hydrocarbons; OBS—oxygenated bicyclic sesquiterpenes; ClevA—Clevenger apparatus; SCom—semi-commercial steam distillation; F—female; M—male.
