# Supplementary material for: Biological Activity of Essential Oils of Four Juniper Species and Their Potential as Biopesticides
Source: Molecules. 2021 Oct 21;26(21):6358. doi: 10.3390/molecules26216358 (PMC8586938; doi:10.3390/molecules26216358)
Supplement: Supplementary file 1 [file molecules-26-06358-s001.zip › molecules-1401771-supplementary.pdf]

Table S1

Constituents and concentrations of *J. communis* extracted using the Clevenger and Commercial extraction methods.

Constituent Number	Volatile constituents	RI	Concentration min-max range (%) Clevenger	Concentration min-max range (%) Commercial
1	α-Thujene	931	0.98 - 1.48	1.01- 1.55
2	α-Pinene	939	26.81 - 35.59	26.48 - 35.34
3	Camphene	953	0.29 - 0.31	0.30 - 0.33
4	Sabinene	969	8.94 - 9.41	9.16 - 9.89
5	β-Pinene	974	1.63 - 1.71	1.67 - 1.80
6	β-Myrcene	991	4.27 - 4.49	4.37 - 4.72
7	β-Fellandrene	1005	0.11 - 0.61	0.12 - 0.64
8	α-Terpinene	1018	0.8 - 0.84	0.82 - 0.88
9	p-Cymene	1025	0.88 - 0.92	0.90 - 0.97
10	Limonene	1031	2.07 - 6.96	2.12 - 7.31
11	γ-Terpinene	1062	1.42 - 1.47	1.45 - 1.54
12	α-Terpinolene	1088	0.92 - 1.09	0.95 - 1.15
13	β-Linalool	1096	0.81 - 1.00	0.83 - 1.06
14	Terpinene-4-ol	1177	3.83 - 4.97	3.93 - 5.22
15	p-Cymene-8-ol	1182	0.18 - 0.85	0.19 - 0.89
16	α-Terpineol	1189	0.71 - 0.74	0.72 - 0.78
17	Bornyl acetate	1285	0.31 - 0.64	0.31 - 0.67
18	α-Cubebene	1351	0.28 - 0.92	0.29 - 0.97
19	α-Copaene	1376	0.49 - 0.66	0.50 - 0.69
20	β-Elemene	1390	0.32 - 1.11	0.33 - 1.17
21	β-Caryophyllene	1419	2.33 - 3.56	2.39 - 3.74
22	γ-Elemene	1433	0.19 - 0.37	0.20 - 0.39
23	α-Humulene	1454	1.29 - 2.63	1.32 - 2.76
24	γ-Muurolene	1479	0.45 - 0.21	0.21 - 0.48
25	Germacrene D	1480	7.27 - 8.57	6.45 - 6.95
26	Elixene	1493	0.92 - 3.29	0.94 - 3.45
27	γ-Cadinene	1513	1.51 - 1.55	1.55 - 1.63
28	δ-Cadinene	1524	4.49 - 6.10	4.12 - 6.41
29	(-)-Spathulenol	1578	1.00 - 2.06	1.03 - 2.16
30	Caryophyllene oxide	1582	2.42 - 2.54	2.25 - 2.64
31	δ-Cadinol	1619	n.d.	0.96 - 1.08
32	tau.-Cadinol	1634	n.d.	0.48 - 0.75
33	tau.-Muurolol	1638	n.d.	0.33 - 0.67
34	α-Cadinol	1641	n.d.	0.73 - 0.95
35	Manoyl oxide	1992	0.63 - 3.52	0.65 - 3.70

n.d. = not detected.

Table S2

Constituents and concentrations of *J. sibirica* extracted using the Clevenger and Commercial extraction methods.

Constituent Number	Volatile constituents	RI	Concentration min-max range (%) Clevenger	Concentration min-max range (%) Commercial
1	α-Thujene	931	1.29 - 1.36	1.32 - 1.42
2	α-Pinene	939	15.38 - 20.51	14.69 - 20.38
3	Camphene	953	0.14 - 0.17	0.14 - 0.17
4	Sabinene	969	13.25 - 14.91	13.52 - 15.56
5	β-Pinene	974	0.57 - 0.66	0.58 - 0.69
6	β-Myrcene	991	1.03 - 1.16	1.05 - 1.21
7	δ-2-Carene	1001	0.79 - 1.26	0.81 - 1.32
8	δ-3-Carene	1007	0.55 - 1.82	0.56 - 1.90
9	α-Terpinene	1018	0.15 - 0.61	0.15 - 0.63
10	p-Cymene	1025	0.49 - 0.61	0.50 - 0.64
11	Limonene	1031	1.20 - 1.23	1.23 - 1.28
12	β-Phelandrene	1033	0.17 - 0.53	0.17 - 0.56
13	trans-β-Ocimene	1050	0.34 - 0.69	0.35 - 0.72
14	γ-Terpinene	1062	1.11 - 1.23	1.13 - 1.28
15	α-Terpinolene	1088	1.18 - 1.33	1.20 - 1.39
16	Terpinene-4-ol	1177	3.41 - 3.54	3.48 - 3.70
17	Bornyl acetate	1285	0.33 - 0.42	0.33 - 0.44
18	Myrtenyl acetate	1298	0.26 - 0.28	0.26 - 0.29
19	α-Terpinyl acetate	1337	0.30 - 0.39	0.31 - 0.40
20	α-Copaene	1378	1.08 - 4.23	1.10 - 4.41
21	β-Elemene	1390	1.02 - 6.59	1.04 - 6.88
22	β-Caryophyllene	1419	1.02 - 1.22	1.04 - 1.25
23	γ-Elemene	1433	0.61 - 5.67	0.62 - 5.92
24	α-Humulene	1454	1.13 - 1.33	1.15 - 1.39
25	Germacrene D	1480	9.75 - 13.65	8.94 - 13.22
26	Elixene	1493	0.61 - 1.84	0.62 - 1.92
27	α-Muurolene	1495	0.55 - 0.78	0.56 - 0.82
28	γ-Cadinene	1513	0.49 - 1.28	0.50 - 1.33
29	δ-Cadinene	1524	5.80 - 6.45	5.91 - 6.73
30	α-Cadinene	1538	0.74 - 0.84	0.76 - 0.88
31	Germacrene B	1556	0.33 - 0.52	0.34 - 0.54
32	trans-Nerolidol	1561	1.95 - 3.99	1.98 - 4.17
33	Germacrene D-4-ol	1575	1.90 - 7.68	1.93 - 8.02
34	δ-Cadinol	1619	0.73 - 0.92	0.75 - 0.96
35	tau.-Cadinol	1634	0.57 - 1.13	0.58 - 1.18
36	tau.-Muurolol	1638	1.25 - 1.33	1.28 - 1.36
37	α-Cadinol	1641	1.44 - 2.85	1.46 - 2.98
38	Farnesol	1692	0.38 - 0.79	0.39 - 0.83
39	Farnesal	1707	0.24 - 0.35	0.25 - 0.37
40	Cembrene	1931	1.19 - 1.22	1.21 - 1.27
41	Totarol	2269	0.47 - 0.54	0.48 - 0.56

Table S3

Constituents and concentrations of *J. pygmaea* extracted using the Clevenger and Commercial extraction methods.

Constituent Number	Volatile constituents	RI	Concentration min-max range (%) Clevenger	Concentration min-max range (%) Commercial
1	α-Thujene	931	1.29 - 2.64	1.31 - 2.69
2	α-Pinene	939	20.99 - 22.18	19.41 - 22.14
3	Camphene	953	0.17 - 0.19	0.18 - 0.20
4	Sabinene	969	15.62 - 25.26	14.94 - 25.36
5	β-Pinene	974	2.24 - 4.4	2.28 - 4.59
6	β-Myrcene	991	1.02 - 1.50	1.04 - 1.57
7	α-Phelandrene	1005	0.41 - 0.77	0.42 - 0.80
8	δ-3-Carene	1007	0.14 - 0.17	0.14 - 0.18
9	α-Terpinene	1018	0.83 - 1.53	0.85 - 1.59
10	p-Cymene	1025	0.96 - 1.24	0.97 - 1.30
11	Limonene	1031	1.43 - 2.08	1.46 - 2.18
12	β-Phelandrene	1033	2.68 - 4.95	2.73 - 5.17
13	γ-Terpinene	1062	1.52 - 2.67	1.55 - 2.79
14	cis-Sabinene hydrate	1068	0.12 - 0.16	0.13 - 0.17
15	α-Terpinolene	1088	1.04 - 1.86	1.06 - 1.94
16	β-Linalool	1096	0.40 - 0.45	0.41 - 0.47
17	trans-Sabinene hydrate	1098	0.20 - 0.22	0.21 - 0.23
18	Borneol	1169	0.31 - 0.37	0.32 - 0.38
19	Terpinene-4-ol	1177	3.19 - 5.83	3.05 - 6.09
20	α-Terpineol	1189	0.40 - 0.60	0.41 - 0.63
21	Myrtenol	1198	0.21 - 0.25	0.22 - 0.27
22	trans-Carveol	1219	0.19 - 0.24	0.20 - 0.25
23	Citronellol	1227	0.23 - 0.26	0.23 - 0.28
24	Bornyl acetate	1285	0.36 - 0.42	0.37 - 0.44
25	Myrtenyl acetate	1298	0.28 - 0.36	0.28 - 0.37
26	α-Copaene	1376	0.30 - 0.51	0.31 - 0.53
27	β-Elemene	1390	1.07 - 4.62	1.09 - 4.83
28	β-Caryophyllene	1419	0.90 - 3.09	0.92 - 3.22
29	α-Humulene	1454	0.52 - 2.89	0.53 - 3.01
30	γ-Muurolene	1479	0.32 - 1.36	0.33 - 1.42
31	Germacrene D	1480	2.43 - 11.06	2.48 - 11.55
32	Elixene	1493	0.70 - 1.41	0.72 - 1.47
33	γ-Cadinene	1513	1.40 - 3.13	1.43 - 3.27
34	δ-Cadinene	1524	2.03 - 5.01	2.08 - 5.23
35	trans-Nerolidol	1561	0.61 - 2.52	0.62 - 2.63
36	Germacrene D-4-ol	1575	0.16 - 1.38	0.16 - 1.44
37	(-)-Spathulenol	1578	0.43 - 1.00	0.44 - 1.05
38	Caryophyllene oxide	1582	1.06 - 2.82	1.08 - 2.94
39	δ-Cadinol	1619	0.24 - 0.49	0.25 - 0.51
40	tau.-Cadinol	1634	0.21 - 0.61	0.21 - 0.63
41	α-Cadinol	1641	1.77 - 2.71	1.45 - 2.83

Table S4

Constituents and concentrations of *J. oxycedrus* extracted using the Clevenger and Commercial extraction methods.

Constituent Number	Volatile constituents	RI	Concentration min-max range (%) Clevenger	Concentration min-max range (%) Commercial
1	Tricyclene	922	0.15 - 0.17	0.15 - 0.18
2	α-Pinene	939	27.84 - 33.79	26.53 - 32.74
3	Camphene	953	0.24 - 0.25	0.25 - 0.27
4	Sabinene	969	0.93 - 1.51	0.95 - 1.60
5	β-Pinene	974	0.44 - 3.81	0.45 - 4.04
6	β-Myrcene	991	0.39 - 3.07	0.40 - 3.26
7	α-Terpinene	1018	0.19 - 0.33	0.13 - 0.35
8	p-Cymene	1025	0.27 - 1.31	0.27 - 1.39
9	Limonene	1031	2.99 - 4.79	3.06 - 5.09
10	γ-Terpinene	1062	0.49 - 0.51	0.50 - 0.54
11	α-Terpinolene	1088	0.47 - 0.50	0.48 - 0.53
12	β-Linalool	1096	0.36 - 0.38	0.37 - 0.41
13	α-Campholenal	1121	1.57 - 1.65	1.61 - 1.75
14	trans-Pinocarveol	1136	0.20 - 0.21	0.21 - 0.23
15	trans-Verbenol	1141	0.28 - 0.29	0.28 - 0.31
16	Borneol	1169	0.17 - 0.18	0.17 - 0.19
17	Terpinene-4-ol	1177	0.31 - 0.33	0.32 - 0.35
18	p-Cymene-8-ol	1182	0.42 - 0.44	0.43 - 0.47
19	Myrtenal	1185	0.70 - 0.73	0.71 - 0.78
20	Myrtenol	1187	0.66 - 0.70	0.68 - 0.74
21	α-Terpineol	1189	0.76 - 0.80	0.78 - 0.85
22	Verbenone	1208	0.44 - 0.46	0.45 - 0.49
23	Bornyl acetate	1285	0.63 - 0.66	0.65 - 0.70
24	Myrtenyl acetate	1298	0.56 - 0.59	0.57 - 0.62
25	α-Copaene	1376	0.78 - 0.82	0.79 - 0.87
26	β-Elemene	1390	0.87 - 0.91	0.89 - 0.97
27	β-Caryophyllene	1419	0.97 - 2.02	0.99 - 2.15
28	γ-Elemene	1433	0.51 - 0.71	0.53 - 0.76
29	α-Humulene	1454	0.33 - 1.41	0.34 - 1.50
30	α-Curcumene	1477	0.89 - 3.30	0.91 - 3.50
31	Germacrene D	1480	3.52 - 17.02	3.60 - 18.06
32	α-Muurolene	1499	0.25 - 0.48	0.26 - 0.51
33	γ-Cadinene	1513	3.87 - 4.24	3.89 - 4.10
34	δ-Cadinene	1524	3.04 - 4.17	2.93 - 4.42
35	α-Cadinene	1537	0.18 - 0.68	0.18 - 0.72
36	α-Calacorene	1549	0.92 - 1.92	0.94 - 2.04
37	trans-Nerolidol	1561	0.81 - 3.81	0.83 - 4.04
38	(-)-Spathulenol	1578	0.53 - 0.95	0.54 - 0.97
39	Caryophyllene oxide	1582	0.77 - 0.87	0.79 - 0.93
40	α-Cedrol	1598	0.41 - 0.87	0.42 - 0.92
41	δ-Cadinol	1619	0.76 - 0.93	0.77 - 0.96
42	tau.-Cadinol	1634	0.80 - 1.50	0.82 - 1.59
43	tau.-Muurolol	1638	0.81 - 0.91	0.83 - 0.97
44	α-Cadinol	1641	0.63 - 0.76	0.64 - 0.80
45	Manoyl oxide	1992	4.03 - 20.18	4.13 - 21.41

Table S5

Main class of compounds in the essential oil (EO) of *J. oxycedrus, J. communis*, *J. pygmaea, J. sibirica.*

Species/ Method/Sex		Class													
		Monoterpenes						Sesquiterpenes						Diterpenes	
		**MH**	**BOM**	**OM**	**BM**	**PhM**	**MOM**	**SH**	**OS**	**TOS**	**BSH**	**OBS**	**ASH**	**MD**	**OD**
J. communis															
**ClevA**	**F**	58.5	-	8.19	-	0.92	-	12.47	-	2.06	10.07	2.42	-	-	0.63
	**M**	53.6	-	5.84	-	0.88	-	11.90	-	1.01	14.06	2.54	-	-	3.52
**SCom**	**F**	58.0	-	8.40	-	0.94	-	11.78	-	2.10	10.32	4.98	-	-	0.65
	**M**	54.0	-	5.98	-	0.89	-	10.02	-	1.03	14.41	5.96	-	-	3.61
J. oxycedrus															
**ClevA**	**M**	36.4	3.60	3.37	-	1.31	-	9.05	-	1.81	15.47	4.87	-	-	20.18
	**F**	46.1	3.42	3.21	-	0.26	-	20.27	-	0.94	13.62	3.87	-	-	4.03
**SCom**	**M**	35.3	3.69	3.46	-	1.35	-	9.28	-	1.86	15.38	4.99	-	-	20.69
	**F**	44.3	3.51	3.28	-	0.27	-	20.78	-	0.96	13.96	3.97	-	-	4.13
J. sibirica															
**ClevA**	**M**	43.6	0.70	3.41	1.33	0.61	5.63	20.45	3.84	-	10.68	4.03	0.62	1.22	-
	**F**	36.6	0.58	3.54	3.08	0.48	7.97	12.53	11.68	-	1.94	6.15	1.15	1.18	-
**SCom**	**M**	43.5	0.72	3.48	1.36	0.62	5.75	19.85	3.92	-	10.87	4.12	0.63	1.24	-
	**F**	36.3	0.59	3.61	3.14	0.49	8.19	11.78	11.91	-	11.16	6.27	1.17	1.21	-
J. pygmaea															
**ClevA**	**M**	67.0	1.16	7.11	0.13	1.24	0.19	4.50	9.29	1.00	5.69	-	-	-	-
	**F**	53.0	1.39	4.25	0.17	0.96	0.24	17.60	5.27	0.43	11.55	-	-	-	-
**SCom**	**M**	65.4	1.19	7.25	0.14	1.27	0.20	4.58	9.47	1.02	5.81	-	-	-	-
	**F**	52.0	1.43	4.14	0.17	0.97	0.24	17.95	5.38	0.44	11.78	-	-	-	-

MH = monoterpenes hydrocarbons; OM = oxygenated monoterpenes; PhM = Phenolic monoterpenes; BM = Bicyclic monoterpenes; MOM = Monocyclic oxygenated monoterpenes; SH = sesquiterpenes hydrocarbons; OS = oxygenated sesquiterpenes; TOS = tricyclic oxygenated sesquiterpenes; BSH = bicyclic sesquiterpene hydrocarbons; OBS = oxygenated bicyclic sesquiterpenes; ASH = Acyclic sesquiterpenes hydrocarbons; D = diterpenes; OD = oxygenated diterpenes.
